# Aberrant ICA and Associated Skull Base Foramina Visualized on Photon Counting Detector CT: Interesting Images

**DOI:** 10.3390/diagnostics15172213

**Published:** 2025-08-31

**Authors:** Ahmed O. El Sadaney, John C. Benson, Felix E. Diehn, John I. Lane, Paul J. Farnsworth

**Affiliations:** Department of Radiology, Mayo Clinic, 200 First Street Southwest, Rochester, MN 55905, USA; rabie.ahmed@mayo.com (A.O.E.S.);

**Keywords:** photon counting detector CT (PCD-CT), energy integrating detector CT (EID-CT), aberrant ICA, persistent stapedial artery (PSA), lateralized ICA

## Abstract

Aberrant internal carotid arteries (ICA) are congenital vascular anomalies that occur from involution of the cervical portion of the ICA, which leads to enlargement of the normally small collateral inferior tympanic and caroticotympanic arteries. The inferior tympanic artery is a branch of the external carotid artery, usually the ascending pharyngeal artery, which extends through the inferior tympanic canaliculus (ITC), a small foramen located along the cochlea promontory. Aberrant ICAs can also be associated with a persistent stapedial artery (PSA), which is an abnormal vessel that arises from the petrous ICA and passes through the obturator foramen of the stapes. An aberrant ICA is a very important anomaly to recognize on imaging. Accurately describing its presence is important to help prevent iatrogenic injury during intervention. It is also important to distinguish an aberrant ICA from a lateralized ICA. The improvement of spatial resolution with photon counting detector (PCD)-CT has been proven to provide higher performance in detection of sub-centimeter vascular lesions compared to conventional energy-integrated detector (EID)-CT. PCD-CT also provides superior visualization of small skull-based foramina such as the inferior tympanic canaliculus, which can aid in more accurately characterizing an aberrant ICA (variant course without ITC involvement).

**Figure 1:**
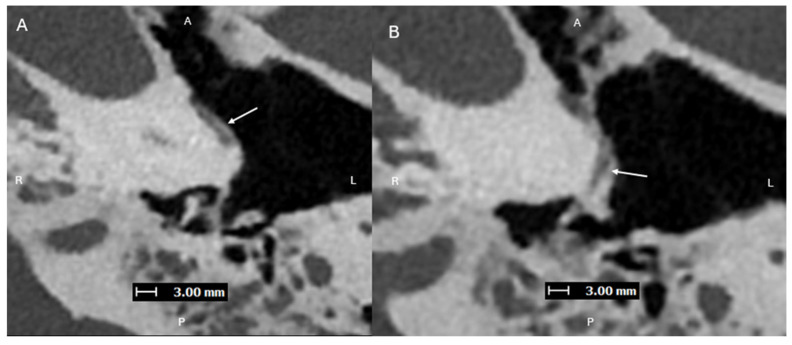
Axial images (superior to inferior (**A**,**B**), with A, P, L, R for orientation donating anterior, posterior, left, and right respectively) from a PCD-CT (Naeotom Alpha; Siemens Healthineers, Forchheim, Germany) temporal bone study—with UHR mode (Collimation 120 × 0.2 mm), Tube voltage (120 kVp), slice thickness/increment (0.2/0.1 mm), and sharp Kernel (Hr84) with Quantum Iterative Reconstruction (3)—demonstrating a normal left inferior tympanic canaliculus ITC (solid arrows) passing between the carotid canal and jugular foramen, traveling along the cochlear promontory. The normally small inferior tympanic artery extends through the ITC and is a branch of the external carotid artery, usually the ascending pharyngeal artery. Normally, the ITC, as in this case, is a thin corticated channel that transmits the tympanic branch of the glossopharyngeal nerve (Jacobson’s nerve) and the inferior tympanic artery. The latter is hard to visualize on EID-CT due to its small size. It can enlarge in case of congenital involution of the cervical portion of the internal carotid artery (ICA), forming the aberrant ICA [1,2].

**Figure 2:**
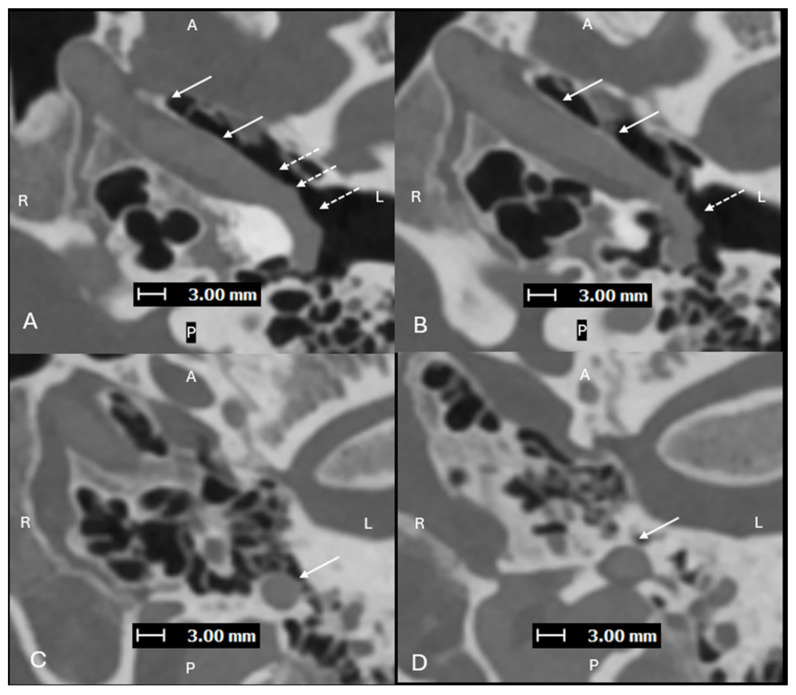
Axial images (superior to inferior (**A**–**D**), with A, P, L, R for orientation donating anterior, posterior, left, and right respectively) from a PCD-CTA study—with UHR mode (Collimation 120 × 0.2 mm), Tube voltage (120 kVp), slice thickness/increment (0.2/0.2 mm), and sharp Kernel (Hv56) with Quantum Iterative Reconstruction (3)—demonstrating an aberrant L ICA (solid arrows) within an enlarged ITC (dashed arrows). Aberrant ICA are congenital vascular anomalies that occur from the involution of the cervical portion of the ICA, which leads to enlargement of the normally small collateral inferior tympanic and caroticotympanic (hyoid) arteries [1]. The segment most noted as the aberrant ICA is the inferior tympanic artery [2]. The inferior tympanic artery is a branch of the external carotid artery, usually the ascending pharyngeal artery, which extends through the inferior tympanic canaliculus (ITC), and is enlarged in the setting of an aberrant ICA [3]. As this artery courses along the cochlear promontory (via the ITC), no carotid plate separates the vessel from the middle ear cavity, and the vessel appears as a vascular retrotympanic mass on exam. It is very important to recognize an aberrant ICA on imaging. Accurately describing the presence of this aberrant vessel is important to help prevent iatrogenic injury during intervention of the middle ear. While typically asymptomatic in some cases, it can present with hearing loss and sometimes pain [4,5]. An aberrant ICA is commonly included in the differential diagnosis for vascular middle ear masses with other entities such as lateralized ICA, dehiscent jugular bulb, aneurysm of the petrous ICA, glomus tympanicum tumors, and glomus jugulare tumors [6,7].

**Figure 3:**
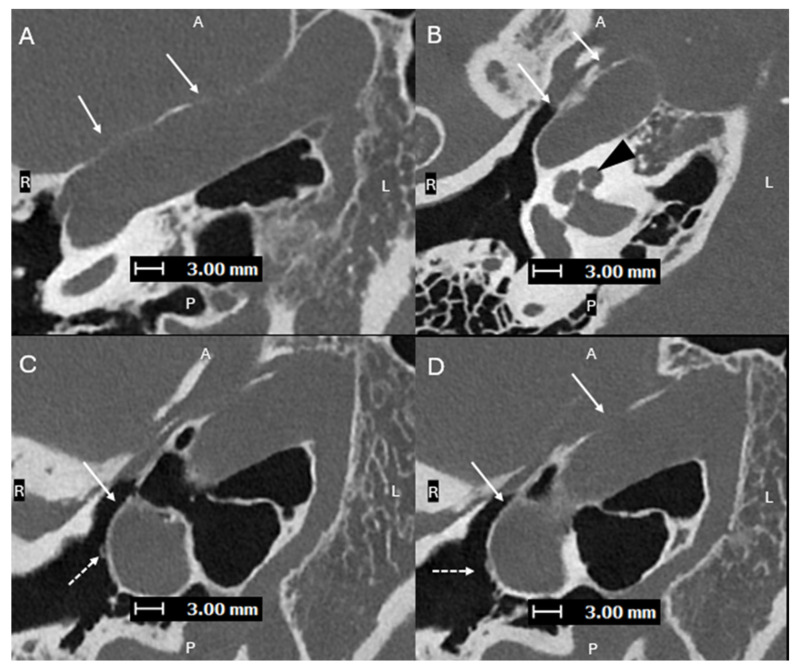
Axial oblique images (superior to inferior (**A**–**D**), with A, P, L, R for orientation donating anterior, posterior, left, and right respectively) from a PCD-CT temporal bone study—with same acquisition and reconstruction parameters as Figure 1—demonstrating a lateralized R ICA (solid arrows). (**B**) shows the ICA lateral to the midportion of the basal turn of the cochlea (black arrowhead). (**C**,**D**) show the normal R ITC visualized laterally (dashed arrows), proving that this is a lateralized ICA and not an aberrant ICA. A lateralized ICA is defined as the genu of the vertical and horizontal segments of the petrous ICA positioned lateral to the midportion of the basal turn of the cochlea in the axial plane. Importantly, the ITC is not affected, which helps to distinguish a lateralized ICA from an aberrant ICA [8]. Skull base foramina, such as the inferior tympanic canaliculus, are much better seen on PCD-CT, which can greatly help when trying to differentiate between an aberrant ICA and a lateralized ICA, as shown in cases 2 and 3 [9]. On EID-CT, it can be difficult to differentiate between a lateralized ICA and an aberrant ICA.

**Figure 4:**
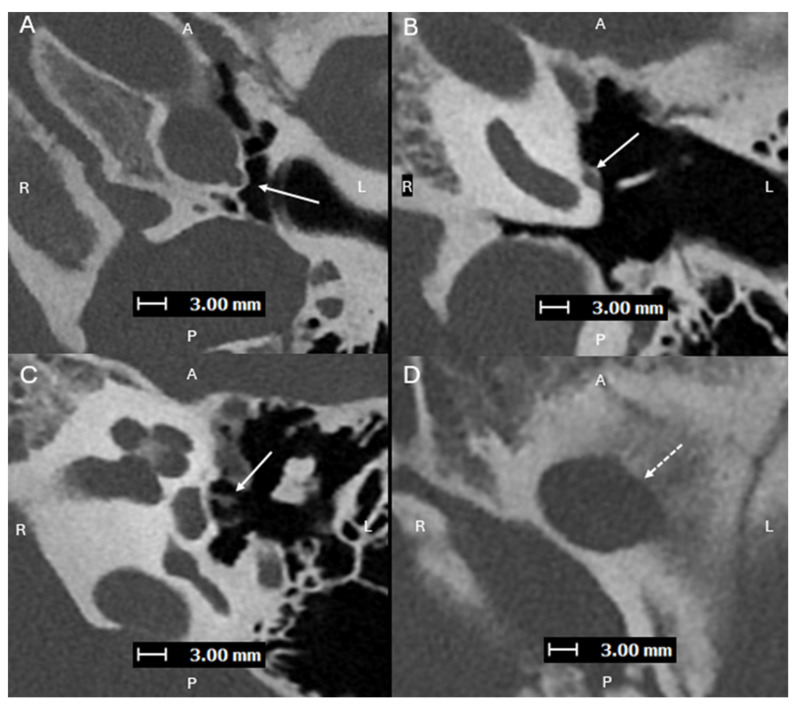
Axial oblique images (superior to inferior (**A**–**C**), with A, P, L, R for orientation donating anterior, posterior, left, and right respectively) from a PCD-CT temporal bone study—with same acquisition and reconstruction parameters as Figure 1—demonstrating a persistent L stapedial artery traveling inferiorly and passing through the obturator foramen of the stapes (**C**). (**D**) shows a normal foramen ovale (dashed line) with absence of the foramen spinosum, which is consistent with a persistent stapedial artery. Aberrant ICAs can be associated with a persistent stapedial artery (PSA), which is an abnormal vessel that arises from the petrous ICA and passes through the obturator foramen of the stapes [10]. The posterior division of the upper stapedial artery branch becomes the middle meningeal artery (MMA). With the normal embryologic atrophy, the distal portion of the MMA is supplied by the internal maxillary artery through the foramen spinosum. If this fails to happen, the territory of the middle meningeal artery will be supplied by the collateral connection with the ophthalmic artery or by a PSA, and absence of the foramen spinosum is expected [11]. A PSA should be recognized prior to stapes surgery. The improvement of spatial resolution with photon counting detector (PCD)-CT has been proven to provide higher performance in detection of sub-centimeter vascular lesions compared to conventional energy-integrated detector (EID)-CT [12].

## Data Availability

The original contributions presented in this study are included in the article. Further inquiries can be directed to the corresponding author.
